# The heart-liver metabolic axis: defective communication exacerbates disease

**DOI:** 10.1002/emmm.201303800

**Published:** 2014-03-12

**Authors:** Kedryn K Baskin, Angie L Bookout, Eric N Olson

**Affiliations:** Department of Molecular Biology, University of Texas Southwestern Medical CenterDallas, TX, USA

## Abstract

The heart has been recognized as an endocrine organ for over 30 years (de Bold, 2011); however, little is known about how the heart communicates with other organs in the body, and even less is known about this process in the diseased heart. In this issue of *EMBO Molecular Medicine*, Magida and Leinwand (2014) introduce the concept that a primary genetic defect in the heart results in aberrant hepatic lipid metabolism, which consequently exacerbates hypertrophic cardiomyopathy (HCM). This study provides evidence in support of the hypothesis that crosstalk occurs between the heart and liver, and that this becomes disrupted in the diseased state.

HCM is an inherited cardiovascular disease primarily caused by mutations in genes encoding proteins in the sarcomere, the contractile apparatus of cardiac myocytes. HCM is characterized by increased heart mass and abnormal cardiac function with susceptibility to arrhythmias and sudden cardiac death. Histological manifestations of the disease include cardiac myocyte hypertrophy, myocardial fibrosis, extracellular matrix disorganization, and myocyte disarray. While many affected individuals are asymptomatic and remain undiagnosed, HCM is the most frequent cause of sudden death in young athletes (Seidman & Seidman, 2011; Maron & Maron, 2013).

To date, 13 genes containing over 900 distinct mutations have been identified as genetic causes of HCM. Most of these genes encode for proteins found within the thick and thin filaments of sarcomeres, such as β-myosin heavy chain (*MYH7*) and troponin T (*TTNT2*). Mutations in *MYH7* increase force generation and actin-myosin sliding velocity within sarcomeres. These findings indicate that genetic mutations in HCM patients are the primary cause of cardiac hypertrophy (Wang *et al*, 2010).

Numerous animal models have been generated to investigate HCM (Maass & Leinwand, 2000), and much focus has been given to an R403Q mutation in *MYH7*, which causes an especially severe clinical phenotype (Seidman & Seidman, 2011). While the various animal models of R403Q highlight different aspects of HCM, they share common traits of HCM including cardiac hypertrophy and fibrosis (Maass & Leinwand, 2000). An interesting, and poorly understood characteristic of hypertrophic cardiomyopathy, as opposed to other types of cardiomyopathies, is that systemic metabolic alterations occur secondary to the cardiomyopathy (Maron & Maron, 2013). This is recapitulated in the R403Q model used in the study published by Magida and Leinwand (2014).

Clinical studies have revealed that HCM patients harboring mutations in sarcomeric genes present with deficient cardiac energetics (Crilley *et al*, 2003). In the present study, the authors demonstrate that the R403Q HCM mouse model has diminished cardiac ATP levels and impaired lipid utilization in the heart, assessed by decreased cardiac triglycerides and fatty acid content, and decreased expression of fatty acid translocase (CD36), lipoprotein lipase (LPL), and very low density lipoprotein receptor (VLDLR). Notably, they observed an approximate two-fold reduction in active CD36 protein at the plasma membrane, coupled with a similarly decreased level of nonesterified fatty acid (NEFA) released from VLDL by the left ventricle. The authors suggest that this decreased lipid uptake in the heart leads to the observed lipid elevation in the plasma, ultimately resulting in hepatic lipid accumulation and pathologically enhanced gluconeogenesis. The authors propose that this elevation in hepatic glucose production creates a vicious cycle between the heart and the liver in which the spillover of VLDL triglyceride and oleic acid from the heart insults the liver via elevated protein kinase C signaling. The liver responds by increasing blood glucose levels leading to exacerbation of the primary cardiac disease (summarized in Fig 1). Importantly, features of the diseased state can be rescued either by restoring the energetic deficit at the level of the cardiomyocyte via AMPK agonism, or by blocking the deleterious elevation in hepatic glucose output using the phosphoenolpyruvate carboxykinase (PEPCK) inhibitor 3-MPA (Magida & Leinwand, 2014).

These findings raise the interesting concept that the lack of use of a specific metabolic substrate by one tissue directly affects another

**Figure 1:**
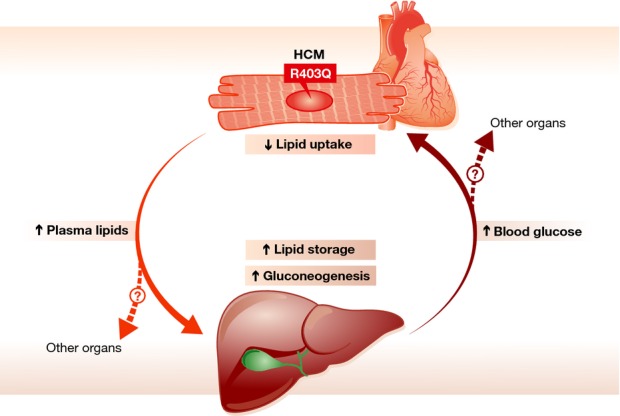
The HCM-causing mutation in myosin (R403Q) decreases cardiac lipid uptake resulting in increased plasma lipid content. Consequently, lipid storage is increased in liver, leading to increased gluconeogenesis, increased blood glucose, ultimately exacerbating cardiac disease. It is still unclear whether other organs are involved in this crosstalk in HCM (denoted in the figure as ‘?’).

These findings raise the interesting concept that the lack of use of a specific metabolic substrate by one tissue directly affects another, perhaps revealing an inter-tissue homeostatic feedback mechanism. Namely, that the heart signals to the liver to elevate glucose production by selectively excluding uptake and use of oleic acid and triglyceride in VLDL particles. Indeed, an emerging theme in homeostatic feedback is the recognition of metabolites as signaling effectors between tissues as means of physiologic integration within the body [see (Blad *et al*, 2012; Liu *et al*, 2013; Roberts *et al*, 2014) for examples]. However, in the setting of an HCM genotype, the current work suggests this relationship is injurious.

Many metabolic diseases, such as diabetes and obesity, are ultimately detrimental to cardiac function, but the reverse has yet to be investigated. There is a clear relationship between cardiac metabolism and cardiac function, but diminished cardiac function, *per se*, has thus far not been reported to negatively influence systemic metabolism. There is a clear link between liver dysfunction, specifically non-alcoholic fatty liver disease, and cardiac dysfunction (Bhatia *et al*, 2012), but new evidence reported in this issue of *EMBO Molecular Medicine* suggests the reverse is also true.

While the link between cardiac dysfunction, specifically the alteration of cardiac metabolism, and deregulated hepatic lipid metabolism is interesting, the mechanisms regulating this crosstalk are not resolved by the work of Magida and Leinwand (2014). Further studies are required to clarify whether HCM-induced metabolic abnormalities are the primary cause of liver dysfunction. It remains unclear whether hepatic lipid accumulation in this mouse model results from decreased fatty acid uptake in the heart alone. Certainly, the relationship between the heart and liver is not monogamous, and other tissues such as skeletal muscle, pancreas, and adipose are likely to be directly affected by elevated circulating oleic acid and VLDL triglyceride. Indeed it is likely that lipid uptake, utilization, or storage in each of these tissues contributes to the metabolic phenotype described by Magida and Leinwand (2014) and would be influenced by systemic agonism of AMPK. Further, PEPCK inhibition not only affects glucose production by the liver, kidney, and intestine, but also glyceroneogenesis in adipocytes. Additionally, it would be interesting to know if other sarcomeric mutations also decrease liver function in end-stage disease, and if so, if a similar mechanism is involved.

Other aspects of HCM can also be explored in the R403Q HCM mouse model within the framework of metabolic abnormalities. For example, what role does calcium homeostasis play in the development of cardiac and metabolic dysfunction? Calcium is an important regulator of energy metabolism and calcium levels and homeostasis are altered in human HCM patients (Wang *et al*, 2010). Perhaps restoring calcium homeostasis in the heart could restore metabolism in this mouse as well? Moreover, what is the basis for the phenotypic gender differences in HCM? Is there likely a protective role for estrogen at the level of cardiac energetics as well as liver metabolism in the HCM patient? Estrogen certainly has a role both as it relates to AMPK and hepatic lipid metabolism (D'Eon *et al*, 2005; Bryzgalova *et al*, 2008), properties which could be therapeutically exploited.

Certainly, the relationship between the heart and liver is not monogamous

The studies of Magida and Leinwand (2014) add to a growing number of examples in which the heart modulates energy homeostasis and metabolism in non-cardiac tissues. In this regard, the cardiac natriuretic peptides, ANP and BNP, have been shown to improve metabolic parameters by inducing the “browning” of white adipocytes (Bordicchia *et al*, 2012). While the thermogenic action by ANP is restricted to human, but not rodent adipocytes (Bordicchia *et al*, 2012), ANP was shown to induce gluconeogenesis in rat hepatocytes (Rashed *et al*, 1992). Therefore, it is curious that ANP expression is dramatically enhanced in HCM, but this mechanism for hepatic glucose output was left unexplored in these studies. Similarly, elevated expression of the Mediator subunit MED13 in the heart confers metabolic benefits in mice. MED13 is negatively regulated by a cardiac specific microRNA, miR-208, which plays a key role in cardiac hypertrophy (Grueter *et al*, 2012). Whether the miR-208/MED13 axis influences the metabolic consequences associated with HCM is an interesting question for the future. Perhaps a miR-208 inhibitor can remedy the metabolic defects observed in HCM by activating cardiac MED13, thus enhancing systemic metabolism, and reversing or preventing liver steatosis.

In summary, the work of Magida and Leinwand (2014) highlights the inextricable relationship between sarcomeric structural integrity and metabolically-derived energy at the organismal level, and opens up many more avenues for future investigation.
